# Translational Pharmacokinetic-Pharmacodynamic Modeling of a Novel Oral Dihydroorotate Dehydrogenase (DHODH) Inhibitor, HOSU-53 (JBZ-001)

**DOI:** 10.3390/pharmaceutics17040412

**Published:** 2025-03-25

**Authors:** Joo Young Na, Min Hai, Kyeongmin Kim, Sandip M. Vibhute, Chad E. Bennett, Christopher C. Coss, Mitch A. Phelps

**Affiliations:** 1 Division of Pharmaceutics and Pharmacology, College of Pharmacy, The Ohio State University, Columbus, OH 43210, USA; na.162@osu.edu (J.Y.N.); hai.29@osu.edu (M.H.); kim.7648@osu.edu (K.K.); coss.16@osu.edu (C.C.C.); 2 Pharmacoanalytical Shared Resource, Comprehensive Cancer Center, The Ohio State University, Columbus, OH 43210, USA; 3 Drug Discovery Shared Resource, Comprehensive Cancer Center, The Ohio State University, Columbus, OH 43210, USA; sandip.vibhute@osumc.edu (S.M.V.); chad.bennett@osumc.edu (C.E.B.); 4 Drug Development Institute, Comprehensive Cancer Center, The Ohio State University, Columbus, OH 43210, USA

**Keywords:** PK/PD modeling, PBPK modeling, first-in-human, HOSU-53, JBZ-001, DHODH inhibitor

## Abstract

**Background:** HOSU-53 (JBZ-001), an orally bioavailable new chemical entity, represents a highly potent dihydroorotate dehydrogenase (DHODH) inhibitor in late preclinical development for application in cancer therapy. **Methods:** Multiple Good Laboratory Practice (GLP) and non-GLP preclinical studies were conducted in mice, rats, and dogs. Plasma samples of HOSU-53 and dihydroorotate (DHO), the substrate of DHODH, were collected for pharmacokinetic (PK) and pharmacodynamic (PD) assessment and modeling. Two modeling approaches were utilized to understand the PK/PD properties of HOSU-53 and to recommend a first-in-human (FIH) dose. **Results:** A population PK/PD model was developed using a stochastic approximation of the expectation-maximization method and evaluated using graphical and numerical methods. The PK of HOSU-53 was well described by a two-compartment model with a first-order absorption and linear elimination, and the PD was described by a turnover model. No covariates were considered significant on PK/PD parameters. This model was subsequently used to predict DHO exposures in humans across a range of doses. Additionally, predicted human hepatocellular HOSU-53 concentrations were obtained from a physiologically based PK model constructed in PK-Sim. **Conclusions:** A first-in-human starting dose of 5 mg once daily was established from the model approaches and will be utilized in the upcoming FIH clinical study.

## 1. Introduction

In rapidly proliferating cells, such as those in growing tumors, the de novo pyrimidine synthesis pathway is highly activated and enhances the cells’ supply of pyrimidine nucleotides, which is a critical survival mechanism for some tumors [1]. Dihydroorotate dehydrogenase (DHODH) is a flavoprotein located in the mitochondrial membrane and facilitates the rate-limiting step in the de novo pyrimidine nucleotide biosynthesis pathway [2,3]. DHODH catalyzes the oxidation of dihydroorotate (DHO) to orotate, which is then converted to uridine monophosphate (UMP), a precursor for cytidine and thymidine biosynthesis [4], by uridine monophosphate synthetase (UMPS) [5,6]. Rapidly dividing cells, including most cancer cells, primarily depend on this de novo pathway, rather than the salvage pathway, which either recycles degraded nucleic acids or imports extracellular nucleosides via nucleoside transporters [3,4,7]. Therefore, targeting de novo pyrimidine synthesis with DHODH inhibitors disrupts the metabolic and biosynthetic processes, leading to the cytotoxic effects observed in tumor cells reliant on this pathway for survival.

DHODH inhibitors are emerging as promising candidates for treating proliferative disorders, including cancers, by stopping tumor cell proliferation in various contexts [8,9,10]. These inhibitors have also been investigated for controlling rapidly dividing immune cells, diseased skin cells, and infectious pathogens [11,12]. Notable FDA-approved DHODH inhibitors include leflunomide and teriflunomide, though they are not approved for cancer indications. Other DHODH inhibitors under investigation as cancer therapies include brequinar, BAY2402234, and Aslan-003, which are no longer under clinical development [13,14]. However, while DHODH remains an attractive target for therapeutic intervention across various clinical conditions, including hematological and solid cancers, significant challenges remain with current compounds. These challenges include poor physicochemical properties, low bioavailability, and limited efficacy or unfavorable safety profiles [15,16,17,18,19,20,21].

HOSU-53 (JBZ-001), an orally bioavailable new chemical entity, is a highly potent DHODH inhibitor in late preclinical development for application in cancer therapy, and it recently received investigational new drug (IND) approval for a first-in-human clinical trial [22]. HOSU-53 demonstrated potent and selective activity against mouse, rat, dog, and human forms of DHODH with low half maximal inhibitory concentration (IC_50_) in cell-free enzyme inhibition assays as a single agent when compared with other clinical candidates mentioned above. This candidate has an acceptable ADME profile, does not induce PXR or inhibit the hERG ion channel, and was negative in both Ames mutagenicity and micronucleus assays. Counter-screening against key safety targets and kinases revealed that HOSU-53′s closest off-target effect is agonizing PPARγ but with a potency 1500× less than it possesses against MOLM-13 AML cells in cell viability assays. HOSU-53 also demonstrated its efficacy against acute myeloid leukemia (AML), multiple myeloma, small-cell lung cancer, melanoma, and other cancer types. Importantly, the in vitro potency of HOSU-53 (0.95 nM IC50 in cell-free human DHODH inhibition assay) was comparable to BAY2402234 (0.97 nM), superior to six other DHODH inhibitors evaluated, and it showed prolonged survival benefits in the MOLM-13 cell line–derived xenograft disseminated AML mice model when combined with other front-line therapies [22].

Given the broad action of DHODH inhibitors across multiple cell types, including rapidly dividing gastrointestinal cells, careful strategies are needed to ensure their safe and effective translation to the clinic. Multiple preclinical pharmacokinetic (PK) and efficacy studies have been carried out on mice, rats, and dogs to help understand the safety and efficacy profiles of HOSU-53 to support further clinical translation. Through the analysis of HOSU-53 PK, the concurrent measurement of plasma DHO levels, and toxicity studies across multiple species, we have identified an exposure level of DHO that, when exceeded and maintained for multiple once-daily oral (PO) dosing intervals, predicts toxicity, and a separate lower level that predicts diminished anti-tumor efficacy [22]. Thus, plasma DHO exposure has been selected as a useful pharmacodynamic (PD) biomarker for toxicity and efficacy for translation from preclinical species into humans. Along the way, attempts to quantify orotate and uridine levels, which could be potential biomarkers, have been explored but discontinued because of method development issues.

Given the rich dataset from preclinical species, we aimed for PK/PD analysis of HOSU-53 to (1) develop population PK/PD and PBPK models of HOSU-53 in different species; (2) quantify the inter-individual variability in PK/PD parameters; (3) evaluate covariates on PK/PD parameters; and (4) extrapolate PK/PD properties to human to aid dose selection in the initial and future human clinical studies.

## 2. Materials and Methods

### 2.1. Animal Studies and Reagents

All animal studies were conducted by external contract research organizations under approved animal use protocols managed by the respective service suppliers, including Charles River Laboratories for mouse studies; Charles River Laboratories, Wuxi AppTec, and Hera BioLabs for rat studies; and Charles River Laboratories and Wuxi AppTec for dog studies. All PO dosing was performed via gavage, and all intravenous (IV) dosing was performed via IV bolus. All reagents and cell lines were supplied by external contractors, except for HOSU-53, which was initially supplied by OSUCCC’s Drug Development Shared Resource and then later by Wuxi AppTec.

During the development of HOSU-53, the chemical formulation was adapted as needed from the free acid to the sodium salt and then the lysine salt for drug substance and drug product development. Initially, HOSU-53 was tested as a free acid in early in vitro experiments. To improve aqueous solubility for in vivo dosing, the sodium salt of HOSU-53 was used for conducting most of the non-GLP in vivo studies. For drug substance and drug product manufacturing, the lysine salt of HOSU-53 was developed and utilized for bridging studies (Supplementary Table S1 Study 7 and 10) and Good Laboratory Practice (GLP) studies (Supplementary Table S1 Study 11 and 12. Bridging PK studies were conducted between sodium and lysine salt, which demonstrated near-identical results. Good Manufacturing Practice (GMP)-grade HOSU-53 (lysine salt form) was provided by STA Pharmaceutical, a subsidiary of Wuxi AppTec, and used for the GLP studies in rats and dogs. All bioanalyses were conducted by the contract laboratories using standard UHPLC-MS/MS analyses. Additional details are provided in Supplementary Materials.

### 2.2. Characterization of HOSU-53 PK and DHO Levels in Mice

We initially evaluated the PK/PD of HOSU-53 in mice in a single-dose study (10 mg/kg, PO; 3 mg/kg, IV) using 40% hydroxypropyl-β-cyclodextrin (HPBCD), the same formulation as in the initial in vivo efficacy studies, to establish early drug exposure and its impact on DHO levels in mice. Subsequently, a single-dose PK/PD study (10 mg/kg, PO; 3 mg/kg, IV) with 20% HPBCD was conducted to reduce the potential impact of formulation components on HOSU-53 disposition. Building on these findings, a repeat-dose PK/PD study in the MOLM-13 human disseminated AML model in male and female triple-immunodeficient NCG mice was conducted. Male mice were dosed at 4, 10, and 20 mg/kg (PO, QD) for 14 days and 30 mg/kg (PO, BIWK) for 15 days, while female mice were dosed at 10 mg/kg (PO, QD) for 14 days (Details in Supplementary Table S1).

### 2.3. Characterization of HOSU-53 PK and DHO Levels in Rats

A series of PK and PD studies was carried out using a range of single and repeat dosing regimens in rats, exploring variations in formulation and dosing schedules. The initial experiment evaluated the PK/PD profile following a single dose of 10 mg/kg PO and 3 mg/kg IV using 40% HPBCD. Building on the single-dose results, repeat dosing was investigated at 1 (both sexes), 3 (both sexes), 6 (females), and 10 (males) mg/kg (PO, QD) and 30 (both sexes) mg/kg (PO, BIWK) regimen in 40% HPBCD formulation. To extend the findings into a disease-relevant context, repeat dosing was evaluated for 14 days in a MOLM-13 xenograft model in the Sprague Dawley Rag2−/− Il2rg−/− (SRG) male rats, where doses of 1, 3, and 10 mg/kg (PO, QD) were administered, alongside 30 mg/kg biweekly (PO, BIWK). A separate experiment examined the PK profile of a lysine salt form of HOSU-53 with a formulation of 50 mM Tris buffer in single doses of 1 and 3 mg/kg (PO) using male and female rats. The GLP experiment focused on a long-term dosing regimen, where male and female rats received oral doses of 1, 2.5, and 5 mg/kg (PO, QD) or 15 mg/kg (PO, BIWK) for 28 days. This phase included a 14-day recovery period to evaluate both chronic exposure effects and reversibility of any observed outcomes (details in Supplementary Table S1).

### 2.4. Characterization of HOSU-53 PK and DHO Levels in Beagle Dogs

The PK/PD characterization of HOSU-53 in beagle dogs was conducted across several studies. An initial single-dose PK/PD evaluation was carried out with male dogs receiving HOSU-53 (10 mg/kg, PO; 3 mg/kg, IV) in 40% HPBCD. Then, repeat-dose PK/PD over 7 days was assessed in male and female dogs with doses of 0.3, 1, and 3 mg/kg (PO, QD) in deionized water. Following that, the lysine salt form was chosen as the preferred salt form, and a single dose bridging PK/PD study was conducted, using 0.3 and 1 mg/kg doses in 50 mM Tris buffer. Additional GLP toxicokinetic and toxicodynamic evaluations were performed, where dogs were dosed at 0.2, 0.6, and 1 mg/kg (PO, QD) for 28 days followed by a 14-day recovery period (Details in Supplementary Table S1).

### 2.5. Population PK Model Development

Population PK analyses were performed using nonlinear mixed-effects modeling implemented in Monolix software (Monolix Suite 2024R1, Antony, France). This approach utilizes the stochastic approximation of the expectation-maximization (SAEM) algorithm coupled with a Markov chain Monte Carlo (MCMC) method to optimize the likelihood [23]. One- and two-compartment PK models incorporating zero- and first-order absorption with linear elimination were evaluated. Interindividual variability (IIV) in PK parameters was assumed to follow a log-normal distribution, except for bioavailability (F), which was modeled using a logit-normal distribution. An exponential model was applied, as described by the following equation:

$$\theta_{i}=\theta_{p}\times e^{\eta_{i}}$$

where $\theta_{i}$ is the model predicted PK parameter for the *i*th animal, $\theta_{p}$ is the typical population PK parameter, and $\eta_{i}$ is the random variable of normal distribution with mean zero and variance $\omega^{2}$.

Residual or random unexplained variability was evaluated using additive, proportional, and combined error models. For each model, several criteria were assessed, including the objective function value (OFV), Akaike Information Criterion (AIC), Bayesian Information Criterion (BIC), corrected Bayesian Information Criterion (BICc), and the precision of parameter estimates, expressed as residual standard errors.

### 2.6. Population PD Model Development

To develop the PK/PD models, a sequential approach was tested for dog data, involving the initial construction of the PK model, fixing the PK parameters with population estimated thetas and eta variances, and then estimating the PD parameters. For mouse and rat data, a simultaneous approach was employed, estimating PK and PD parameters concurrently, assuming log-normal distribution for both. To account for the delay in PD response that results from the inhibition of DHODH, the factor controlling the dissipation of the response, various modeling approaches were employed. One approach involved using an effect compartment model with an equilibrium half-life parameter ($k_{e0}$) to characterize the delay,

For example, effect compartment 4,

$$\frac{dA\left(4\right)}{dt}=k_{e0}\cdot \left(\frac{A\left(2\right)}{V2}-A\left(4\right)\right)$$

where $\frac{dA\left(4\right)}{dt}$ represents the rate of change of the drug effect compartment concentration *A*(4) over time, *A*(2) represents the amount of drug in the central compartment, and *V*(2) represents the apparent volume of distribution of the central compartment.

Another approach was an indirect response model, which allowed for distinct rate constants to describe the onset and offset of the response [24].

These approaches increase the complexity of the PK/PD model, making parameter estimation using the first-order conditional estimation method with interaction (FOCE-I) more challenging [23]. This provided an additional opportunity to construct the model using the SAEM algorithm, implemented in Monolix, to improve parameter estimation and model stability.

### 2.7. Covariate Analysis

Covariates were evaluated in the final PK/PD models using a stepwise covariate selection process. Statistical significance was determined by a reduction in the OFV of 3.84 (1 degree of freedom, *p* = 0.05) for forward selection and 5.99 (1 degree of freedom, *p* = 0.01) for backward elimination. Given the scientific interest in assessing the impact of different salt forms and formulations of HOSU-53, which were administered to animals to enhance solubility and absorption, these factors were included as covariates in the population PK analysis. Additionally, dose level and sex were considered in the covariate analysis for PK/PD. The effect of each categorical covariate was modeled using the following approach:

$$\theta_{i}=\theta_{p}\cdot \left(1+\theta_{k}\cdot \beta \right)\cdot \exp\left[\eta_{i}\right]$$

where $\theta_{i}$ is the model predicted PK/PD parameter for the *i*th animal, $\theta_{p}$ is the typical population PK/PD parameter, $\eta_{i}$ is the random inter-individual variability, β is an indicator variable for a categorical covariate, and κ is an index for the κth covariate effect.

### 2.8. Model Evaluation

The predictive performance of the model was assessed through diagnostic plots, including goodness-of-fit (GOF) plots, residual distribution plots, observed vs. predicted plots, and prediction-corrected visual predictive check (pcVPC) plots. The precision of parameter estimates, expressed as relative standard errors (RSEs), was also evaluated. Additionally, a bootstrap analysis was conducted using Monolix 2024R1 to further assess parameter precision and model robustness.

### 2.9. PBPK Model Development

PBPK models were developed using PK-Sim v11.0 in rats and beagle dogs, and each was used to predict data in the other species, including mice. A base model was parameterized using lipophilicity, plasma protein binding, thermodynamic solubility, and pKa measured from in vitro studies. Half-life of HOSU-53 measured in beagle cryo-preserved hepatocytes was used along with hepatocyte incubation density within the In Vitro to In Vivo Extrapolation (IVIVE) tools in PK-Sim to estimate metabolic clearance in vivo [25]. Renal clearance of unbound plasma HOSU-53 was assumed to be driven solely by glomerular filtration rate (GFR). Various methods were used to calculate partition coefficients and cellular permeabilities in PK-Sim. PK predictions generated for each combination of calculation methods were compared to the mean observed IV profile, and the methods that best matched the observed data were selected. Ultimately, partition coefficients were calculated using the Rodgers and Rowland method [23], and cellular permeabilities were calculated using the charge-dependent Schmitt method [24]. PK following oral administration of HOSU-53 was used to identify parameter values for intestinal permeability for the sodium and lysine salt forms administered in deionized (DI) water. The final dog PBPK model was validated by comparing observed PK data with model predictions following the oral administration of HOSU-53 in a virtual beagle population of 100 animals with body weights ranging from 6 to 9 kg. Similarly, the final rat PBPK model was validated by comparing observed PK data with model predictions following the oral administration of HOSU-53 in a virtual rat population of 100 animals with body weights ranging from 200 to 300 g. The input parameters for the final PBPK model are presented in Table 1 and Supplementary Table S2 for dogs and rats, respectively.

### 2.10. Extrapolation of PBPK Model to Predict Human PK

The final preclinical PBPK models were extrapolated to humans. Partition coefficient and cellular permeability were unchanged from the source species. Half-life of HOSU-53 clearance measured in human cryo-preserved hepatocytes was used along with hepatocyte incubation density within the IVIVE tools in PK-Sim to estimate metabolic clearance in vivo [25]. Values for blood to plasma partition coefficient and plasma protein binding were allowed to vary within the range based on in vitro experiments and predicted for preclinical models. IC_90_ for inhibition of DHODH was determined from in vitro cell-free DHODH inhibition assay to be 669 pM, which was subsequently used as a target for steady-state intracellular hepatocyte concentrations, as DHODH is primarily expressed in liver tissue in healthy human populations [26,27]. The final extrapolated human model was then used to identify a dose of HOSU-53 that would lead to average steady-state liver trough concentration of unbound (free) HOSU-53 being higher than the target 669 pM concentration. Additionally, the final PBPK model was used to simulate the plasma concentration of HOSU-53 every minute for up to 360 h following single and multiple dose administrations in a human virtual population with parameters shown in Table 2.

### 2.11. Human PD Prediction

Both PBPK and population PK model-predicted human plasma concentration-time profiles of HOSU-53 were used to predict the PD profiles of DHO in humans. The human PD profile by time was calculated using the following equations:

$$\frac{dR}{dt}=k_{in}-k_{out}\cdot \left(1-\frac{I_{max}\cdot C^{\gamma }}{{{IC}_{50}}^{\gamma }+C^{\gamma }}\right)\cdot R$$

where *R* is the response level, *C* is the plasma HOSU-53 concentration by time (t), $k_{in}$ is the input rate of the response and was calculated as $R_{0}\times k_{out}$, *I_max_* is the maximum inhibitory effect of the drug on the response, *IC*_50_ is the half-maximal inhibitory concentration, and *k_out_* is the degradation rate.

The area under the curve of DHO (AUC_DHO_) was then calculated using the linear trapezoidal method. Based on the in vitro experimental results, the target AUC_tau,ss_ value for DHO was set to be less than 1000 µM∙h.

## 3. Results

### 3.1. Study Population Characteristics

From the three mouse studies, a total of 309 PK samples and 253 PD samples from 45 mice were included for PK/PD model development (3 mg/kg = 9, 4 mg/kg = 9, 10 mg/kg = 27). None of the PK samples were below the limit of quantification excluding the pre-dose samples. Out of a total of 253 PD samples, only 9 samples (less than 4%) were below the limit of quantification and were excluded from analysis.

From the five rat studies, a total of 949 PK samples and 951 PD samples from 121 rats were included for PK/PD model development (1 mg/kg = 30, 2.5 mg/kg = 12, 3 mg/kg = 21, 5 mg/kg = 12, 6 mg/kg = 3, 10 mg/kg = 15, 15 mg/kg = 12, 20 mg/kg = 4, 30 mg/kg = 12). One of the PK samples was below the limit of quantification, excluding the pre-dose samples. Out of a total of 951 PD samples, 49 samples were below the limit of quantification and were excluded from the analysis.

From the four dog studies, a total of 507 PK samples and 431 PD samples from 39 dogs were included for PK/PD model development (0.2 mg/kg = 8, 0.3 mg/kg = 12, 0.6 mg/kg = 10, 3 mg/kg = 9). None of the PK samples were below the limit of quantification, excluding the pre-dose samples. Out of a total of 431 PD samples, including the pre-dose samples, 92 samples (21.3%) were below the limit of quantification and were excluded from analysis. More information about the datasets used can be found in Supplementary Table S1.

All dogs in the 28-day GLP study survived until scheduled euthanasia. In females treated with 1 mg/kg (PO, QD), lower heart rate and longer RR interval duration were observed at 1–2 h postdosing during weeks 1 and 4. In both males and females treated with 1 mg/kg (PO, QD), adverse effects were observed, which included decreased food consumption, lower body weights, and adverse intestinal microscopic findings related to mucosal epithelial cell loss with correlating macroscopic findings of dark red discoloration. We therefore excluded the 1 mg/kg (PO, QD) data from the modeling and other data analyses.

### 3.2. PK and Safety Profiles in Preclinical Studies

Non-clinical PK of HOSU-53 has been studied and analyzed after single and multiple doses with noncompartmental analysis (NCA). After a single IV (3 mg/kg) and oral (10 mg/kg) dose administration, the time course of HOSU-53 concentration in plasma was similar in rats and dogs, with a fast absorption followed by a mean elimination half-life (T1/2) of 6 to 8 h. F was similar between the two species, with mean values of approximately 60% and 50% in rats and dogs, respectively. In mice, the PK of HOSU-53 was characterized by a higher F of 86% but lower clearance, leading to a longer mean T_1/2_ of 30 h. After repeated oral dosing, HOSU-53 levels in plasma increased in a dose-proportional manner at lower doses in mouse and rat but in a less-than-dose-proportional manner at higher doses in mice (dose range: 4–30 mg/kg) and rats (dose range: 3–10 mg/kg). Concentrations increased in a dose-proportional manner in dogs (dose range: 0.3–3 mg/kg). The rate of distribution of HOSU-53 from plasma, however, remained similar, and the T_1/2_ remained unchanged after repeated oral dosing and did not seem to change with increasing doses.

In both male and female rats, adverse effects associated with elevated DHO exposure at 5 mg/kg (PO, QD) included immune system-related effects, such as decreased lymphoid cellularity in the thymus, mild-to-moderate decreases in lymphocyte counts, and lower thymus weights. These findings suggest that prolonged DHODH inhibition and subsequent pyrimidine depletion may impair immune function. In both male and female dogs, adverse effects associated with elevated DHO exposure were observed at 1 mg/kg (PO, QD), primarily affecting the gastrointestinal (GI) system and metabolism. Observed toxicities included decreased food consumption, lower body weights, and adverse intestinal (colon, ileum, and/or jejunum) microscopic findings related to mucosal epithelial cell loss with correlating macroscopic findings of dark red discoloration. These GI toxicities are consistent with the high proliferative demand of intestinal epithelial cells, which rely on pyrimidine availability for DNA synthesis and repair. The No Observed Adverse Effect Levels (NOAELs) from the rat and dog GLP toxicity studies were then determined to be one dose level lower, which are 2.5 mg/kg (PO, QD) and 0.6 mg/kg (PO, QD), respectively. Based on repeat dose studies in rats, dogs, and tumor-bearing mice, efficacy, tolerability, and toxicity can be correlated with levels of the PD biomarker DHO in plasma. Severe toxicities were observed in all species when the plasma AUC_tau,ss_ of DHO exceeded 1000 μM·h.

### 3.3. Population PK Model

The final structural PK models for both mouse and dog data were two-compartment disposition models with first-order absorption and linear elimination. We ultimately chose to exclude rat data from the population modeling effort, as described below in this section. The parameters for the final dog and mouse PK models are presented in Table 3 and Supplementary Table S3, respectively. All parameters were estimated with good precision, as indicated by the %RSE of the estimates. Proportional and combined error models were selected for the mouse and dog data, respectively.

**Figure 1:**
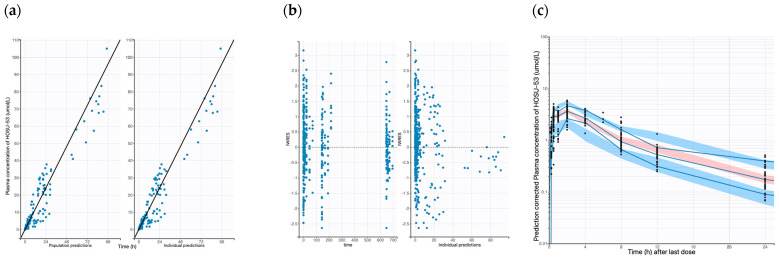
Goodness-of-fit plots of the final population PK dog models. (**a**) Observed HOSU-53 concentration vs. population predicted and observed HOSU-53 concentration vs. individual predictions. (**b**) IWRES vs. time and individual predictions. (**c**) Predicted-corrected visual predictive check (pcVPC) for the final population PK model of dog (Solid blue lines represent 5th, median, and 95th percentile for observed data. Blue areas represent 95% CIs for 5th and 95th percentile of simulated data. Red area represents 95% CIs for median of simulated data).

During the development of the PK model for dogs, a two-compartment model was first constructed using IV data alone. High variability was observed in the pooled IV and PO dataset, and thus, four PK parameters (V1, CL, Q, and V2), IIV on CL, and residual errors were fixed to achieve estimates of bioavailability (F) and absorption rate constant (Ka). This approach provided reasonable estimates for F and Ka, with acceptable RSE values (Table 3). Inspection of the plots of individual random effects against covariates showed no discernible trends for both the mouse and dog models. Furthermore, none of the covariates tested on the PK parameters were found to be significant, indicating that their inclusion did not improve the fit of either model. The GOF plots for the final PK models of dog and mouse are presented in Figure 1 and Supplementary Figure S1, respectively.

The plots of population-predicted versus observed HOSU-53 concentrations, as well as individual-predicted versus observed HOSU-53 concentrations, displayed a symmetric distribution around the line of identity, indicating a strong correspondence between observed and predicted concentrations and good model fit for both the mouse and dog PK models. The observed and predicted trends are closely aligned, though some deviation is observed in the middle portion of the time course, as seen in the data between 6 and 12 h after the last dose. Overall, these GOF plots indicate the final model appropriately describes the PK data with no obvious bias or model misspecification. PcVPC plots for the final dog and mouse models are shown in Figure 1c and Supplementary Figure S1, respectively. The pcVPC plots showed that the predicted median curve obtained by simulation demonstrated an adequate fit to the observed data for both mice and dogs. The nonparametric bootstrap estimates (medians) of the parameters, along with their 95% confidence intervals (CIs), were close to the respective values obtained from the original dataset (Table 3, Supplementary Table S3).

Supplementary Figure S3 illustrates the PK data for all dose levels of HOSU-53 concentrations over time in rats. Each color of the data points represents a specific dose level, as indicated in the legend. Notably, the lack of consistent proportionality between dose levels and normalized PK profiles is evident. While some dose levels appear to overlap or exhibit similar trends in normalized concentrations, others show significant deviations, particularly at higher doses, where variability and divergence become more pronounced. This variability suggests a nonlinear relationship between dose and exposure, which hindered our ability to build a reliable rat PK model, as the foundational assumption of dose-proportionality is not met.

### 3.4. Population PK/PD Model

The final PK/PD model for both species was an indirect turnover model with degradation inhibition. The parameters for the final dog and mouse PK/PD models are presented in Table 3 and Supplementary Table S3, respectively. The dynamics of DHO in plasma were described as follows:

$$\frac{dR}{dt}=k_{in}-k_{out}\;\cdot \;\left(1-\frac{{C_{p}}^{\gamma }}{{C_{p}}^{\gamma }+{{IC}_{50}}^{\gamma }}\right)\cdot R$$

where *R* is the plasma DHO level, $C_{p}$ is the concentration of HOSU-53, ${IC}_{50}$ is the half-maximal inhibitory concentration, $k_{out}$ is the degradation rate, and $k_{in}={R_{0}\times k}_{out}$, $R_{0}$ being the initial (steady-state) value of plasma DHO.

**Figure 2:**
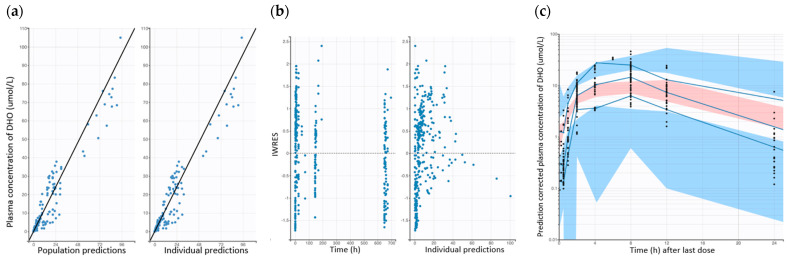
Goodness-of-fit plots of the final population PD dog models. (**a**) Observed DHO concentration vs. population predicted and observed DHO concentration vs. individual predictions. (**b**) IWRES vs. time and individual predictions. (**c**) pcVPC for the final population PD model of dog (Solid blue lines represent 5th, median, and 95th percentile for observed data. Blue areas represent 95% CIs for 5th and 95th percentile of simulated data. Red area represents 95% CIs for median of simulated data).

The GOF plots for the final PD models of dogs and mice are presented in Figure 2 and Supplementary Figure S2, respectively. The plots comparing population-predicted to observed DHO concentrations, as well as individual-predicted to observed DHO concentrations, exhibit a symmetric distribution around the line of identity. This indicates a good correlation between observed and predicted concentrations, demonstrating an acceptable and overall good model fit for both the dog and mouse PD models. Overall, the GOF plots and pcVPCs demonstrate the final model adequately captures the PD data, with no obvious indication of model misspecification (Figure 2 and Supplementary Figure S2).

### 3.5. Human PKPD Prediction

**Figure 3:**
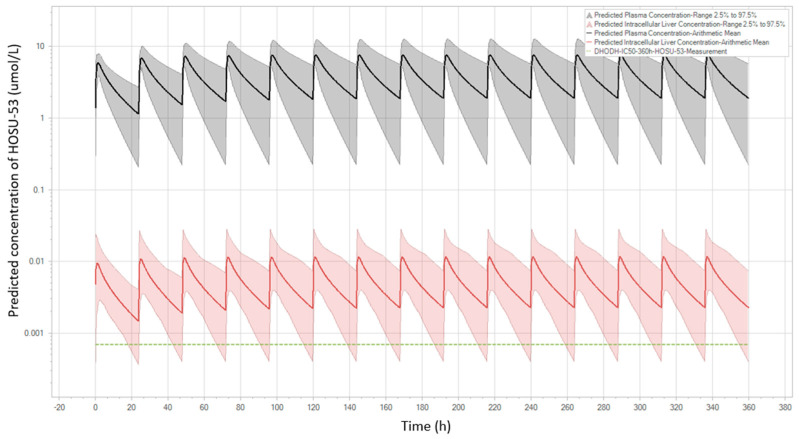
Predicted PK profiles of HOSU-53 in human using final PBPK dog model. The black line with the grey shaded area is the projected plasma level with a 95% predictive interval. The red line with the pink shaded area is the projected hepatic intracellular level with a 95% predictive interval. The green dashed line is targeted efficacious hepatic intracellular levels.

The final PBPK models of rats and dogs were developed using the dataset of HOSU-53 after IV and PO administrations (Supplementary Figures S4 and S5). The final dog PBPK model was applied to predict human PK profiles of HOSU-53 (Figure 3) and compared to predicted human profiles from the final rat PBPK model (Supplementary Figure S6). A human virtual population consisting of 100 subjects (50% female) between the ages of 25 to 80 years and a BMI of 18 to 32 kg/m^2^ was created for the final simulations. Estimated blood to plasma partition coefficients from rats and dogs were in close agreement (0.59 versus 0.61). Plasma protein binding for HOSU-53 varied uniformly within the virtual population, with the free fraction ranging from 0.14 to 0.7%. This range was based on results from an in vitro serum shift assay, whereby the shift in HOSU-53 cytotoxicity in MOLM-13 cells was used to estimate binding to human serum proteins, utilizing methods similar to those previously described [28,29] . This approach was utilized since low free/unbound HOSU-53 could not be accurately measured in protein binding assays. To account for potential differences in intestinal absorption across species and salt forms, intestinal permeability was assumed to be uniformly distributed, 0.004 to 0.07 cm/min based on parameter estimation from PBPK model. Using both the dog and rat PBPK models, simulations were performed at dose levels from 5 to 25 mg HOSU-53 administered once daily for 7 days. The simulation results showed that at a dose level of 25 mg once daily, trough liver concentrations of HOSU-53 were above target free concentrations (669 pM) for 80% of time for 50% of the population. This dose was considered an efficacious dose for HOSU-53 (Table 4).

**Figure 4:**
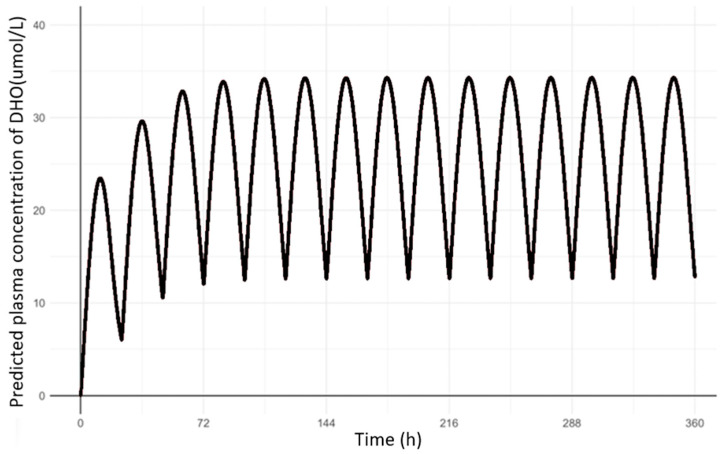
Predicted PD (DHO) profiles in human after 25 mg once daily oral administrations of HOSU-53.

Based on the simulated human PK profiles of HOSU-53, PD parameters from the final dog population PD model were utilized to predict PD profiles in humans after oral administrations of 25 mg once daily (Figure 4). The AUC_tau,ss_ of DHO was calculated to be 623 µM·h, which was within the target range of 1000 µM·h.

## 4. Discussion

HOSU-53 is a novel compound that binds with high affinity to DHODH, effectively inhibiting de novo pyrimidine synthesis, a critical process for rapidly proliferating cancer cells. This inhibition induces pyrimidine starvation, which can result in terminal differentiation, cell cycle arrest, and apoptosis. Previously, HOSU-53 exhibited potent and selective activity against human, mouse, rat, and dog DHODH in enzyme inhibition assays [22]. Plasma DHO levels, the substrate of the DHODH enzyme, serve as a direct PD indicator of HOSU-53′s inhibition of DHODH, providing valuable insights into its efficacy, tolerability, and toxicity. Studies revealed that repeated high doses of HOSU-53 led to DHO accumulation in both rat and dog models, correlating with observed toxicity, whereas no DHO accumulation was detected at lower dose levels following multiple administrations. We have identified the area under the plasma DHO concentration-time curve (AUC_tau,ss_) as a key determinant of HOSU-53′s safety and efficacy in preclinical animal models (Supplementary Figure S7). Consequently, plasma DHO levels were selected as the PD marker in our PK/PD model to optimize dose selection and therapeutic outcomes for preclinical studies and future human clinical trials.

Here, we reported population PK/PD and PBPK models developed from multiple PK/PD studies conducted in mice, rats, and dogs to characterize and assess the efficacy and toxicity of HOSU-53 as a single agent. This represents the first modeling approach integrating all these features, enabling the comparison of PK and PD properties of HOSU-53 across different species. Unlike previous models that primarily focused on empirical PK relationships, our approach incorporates delayed PD effects using an indirect turnover model with degradation inhibition, which mechanistically describes the reduction in orotate degradation due to DHODH inhibition. Both population PK/PD and PBPK models demonstrated acceptable predictability both within and between species. The population PK/PD model indicated dose levels, sex, drug formulation, and salt form were not significant covariates affecting the PK/PD parameters of HOSU-53. Estimated PD parameters within the population PD models for mice and dogs showed overall comparability between the two species. Considering that HOSU-53 is a DHODH inhibitor, which slows the conversion of DHO to orotate thus reducing its degradation rate, an indirect turnover model with degradation inhibition was evaluated and deemed adequate to explain the PD properties of HOSU-53. By incorporating both population PK/PD and PBPK models, this approach enhances predictive accuracy and provides a framework for optimizing dose selection and clinical translation of HOSU-53 as a DHODHi for use in cancer based on preclinical studies. Our combined methodology and indication are, to our knowledge, unique in DHODH inhibitor development.

From a comprehensive series of toxicology studies for HOSU-53 conducted in rats and dogs, including GLP-compliant 28-day repeat-dose toxicokinetic studies with 14-day recovery period in both species, the NOAELs for once-daily administration were determined to be 2.5 mg/kg in rats and 0.6 mg/kg in dogs, where HOSU-53 was well tolerated in both species. Adverse effects associated with HOSU-53 were generally mild to moderate and resolved upon discontinuation of treatment. Following FDA guidance [30] to estimate the FIH dose, the highest non-severely toxic dose (HNSTD) or severely toxic dose for 10% of animals (STD10) was determined to be 5 mg/kg/day in rats and 0.6 mg/kg/day in dogs, which corresponds to 0.8 mg/kg/day human equivalent dose (HED) scaled by body surface area in rats (divided by 6.2) and 0.3 mg/kg/day HED scaled by body surface area in dogs (divided by 1.8), respectively. After applying a safety factor of 10-fold for both species and taking the average, a proposed starting dose of 5 mg flat dose in a 70 kg human was recommended. Based on this suggestion, an FIH study will be conducted with a starting dose of 5 mg. Also, based on PBPK model predictions, which estimated a human dose of 25 mg, the highest dose for the upcoming first-in-human study was set at 25 mg. Based on our preclinical experiments, the optimal efficacy is expected at an AUC_tau,ss_ value of DHO around 300 µM·h, with a therapeutic window ranging from 300 to 1000 µM·h. Our current simulation data for a 25 mg oral dose administered once daily suggest an exposure of 623 µM·h, which falls within the therapeutic range.

While it is not so useful to compare HOSU-53 PK predictions with observed clinical data of other DHODH inhibitors, we have provided some analysis of our predictions of DHO dynamics relative to the limited data that is published in the literature regarding DHO dynamics in both plasma and urine. From the dog PKPD model, the anticipated maximum human plasma DHO concentrations are ~791 ng/mL and ~5500 ng/mL with 5 mg and 25 mg doses in humans, respectively. The literature is sparse with DHO data obtained in humans receiving DHODH inhibitors, but what is available indicates a max of 900 ng/mL is safe (with BID dosing for 7 days with RP7214) [31], and there are no DHO concentrations presented that are unsafe. In our preclinical toxicokinetic data, we observe no toxicity, with maximum DHO concentrations up to ~5850 ng/mL in dogs and ~2870 ng/mL in rats. One publication indicated safety in mice up to ~25 μM DHO (or ~3200 ng/mL, with single dose BAY2402234) [32]. There is no published data available that we have found providing AUC levels for DHO in humans who are receiving DHODH inhibitors.

There are several limitations in this study. First, PBPK modeling is reported to be associated with a high probability of mispredicting PK parameters, which may cause bias in extrapolating preclinical data to humans [33]. However, considering that PBPK is still yielding higher fractions of reliable predictions than traditional allometry, this approach proves to be valuable [33]. However, considering that PBPK still yields higher fractions of reliable predictions than traditional allometry, this approach remains valuable [33]. Secondly, a population PK/PD model for rats was not developed due to the absence of dose proportionality. Unlike in mice and dogs, dose proportionality was not observed in rats, which may be attributed to the limited number of animals or potential procedural inconsistencies. Further analysis is required to investigate this issue. Lastly, our mouse data showed relatively longer elimination half-life compared to rats and dogs in NCA results. One potential contributor to the longer half-life observed in mice is that protein binding may have been substantially higher than in the other two species. Since protein binding was difficult to measure accurately using available methods, as described herein, this is a feasible explanation. While we did explore this possibility within our models, we note that this did not fully explain the PK differences in mice compared to rats and dogs. Given the low overall impact of this difference on our confidence in our dose projections, we have not conducted additional investigations into the source of the murine discrepancy.

**Figure 5:**
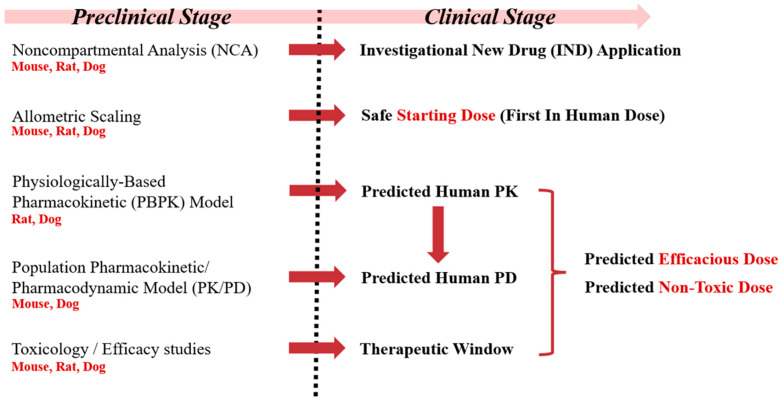
Conceptual framework of preclinical to clinical drug development of HOSU-53.

Considering that leflunomide and teriflunomide are currently only approved for rheumatological indications [34], our current approach represents a comprehensive way to derive dosing strategies to effectively and safely modulate DHODH with HOSU-53 in the context of cancer utilizing plasma drug levels and DHO, which will benefit other DHODH inhibitor development. Also, this study integrated multiple GLP and non-GLP preclinical studies across three species, ensuring a thorough understanding of HOSU-53′s PK and PD. Utilizing population PKPD model and PBPK model enhanced the accuracy of human dose predictions. The modeling approaches provided mechanistic insights by linking plasma DHO levels to HOSU-53 exposure, reinforcing its pharmacological action (Figure 5). Our study highlights the importance of modeling approaches in optimizing drug development, ensuring a data-driven transition from preclinical to clinical phases while minimizing any potential risks. Overall, our study shows how advanced modeling approaches can improve the safety and success of early-phase oncology drug development.

## 5. Conclusions

This study established population PK/PD and PBPK models which provided reasonable agreement for predicted PK (plasma HOSU-53 profiles) and PD (plasma DHO profiles) across species. Using these modeling approaches, the PK and PD profiles of HOSU-53 in a human population were predicted, enabling the determination of the target dose and starting dose for human clinical studies. Considering that HOSU-53 is still under development, refining these models with additional data can further support evidence-based dosing strategies.

## Figures and Tables

**Table 1:** Input parameters for the final dog PBPK model for HOSU-53.

Parameter	Value (Unit)	Source
Lipophilicity	4.35	Experimental data
Fraction unbound in plasma (Fupls)	0.26%	Estimated in PKsim
pKa	1.19 (Base)	Experimental data
	3.05 (Acid)	Experimental data
Solubility	18.8 μg/mL (pH 6.5)	Experimental data
Intestinal Permeability (Sodium Salt—DI Water)	0.008 cm/min	Estimated in PKsim
Intestinal Permeability (Lysine Salt—DI Water)	0.02 cm/min	Estimated in PKsim
Specific Clearance	8.51 cm/min	Scaled from in vitro hepatic stability assay
Blood:Plasma Concentration Ratio	0.61	Estimated in PKsim

**Table 2:** Key parameters of the virtual human population used in PBPK model simulation.

Parameter	Values
Number of Subjects	100
% female	50
Age	25–80 years
Weight	45–120 kg
BMI	18–32 kg/m^2^
Fraction Unbound in Plasma	Distribution: Uniform Minimum: 0.14% Maximum: 0.70%
Intestinal Permeability	Distribution: Uniform
	Minimum: 4 × 10^−3^ cm/min Maximum: 7 × 10^−2^ cm/min
Blood:Plasma Partition Ratio	Distribution: Log-normal
	Mean: 0.61
	Geometric Standard Deviation: 1.20

**Table 3:** PK parameter estimates of the final dog PKPD model.

Parameters	Model Estimates (RSE%)	Bootstrap Result	
		Point Estimate	95% CI
F (%)	0.67 (2.8)	0.67	0.63–0.7
Ka (/h)	1.53 (12.6)	1.55	1.28–1.77
CL/F (mL/h)	150 Fix		
V1 (mL)	980 Fix		
Q (mL/h)	400 Fix		
V2 (mL)	490 Fix		
IIV F	0.24 (60.1)	0.17	0.08–0.31
IIV Ka	0.59 (20.1)	0.59	0.5–0.69
IIV CL	0.2 Fix		
Additive residual error (PK)	0.0033 Fix		
Proportional residual error (PK)	0.45 Fix		
R0 (μmol/L)	0.06 (1.6)	0.05	0.03–0.07
K_out_ (/h)	52 (11.4)	70	44–113
IC_50_ (μmol/L)	0.1 (10.3)	0.08	0.06–0.11
gamma	1.9 (1.6)	1.86	1.69–2.1
IIV K_out_	0.42 (14.9)	0.4	0.28–0.46
IIV IC_50_	0.44 (12.9)	0.42	0.3–0.52
Proportional residual error (PD)	0.55 (5.1)	0.55	0.52–0.58

Abbreviations: F, bioavailability; Ka, first-order absorption rate constant; CL, clearance; V1, volume of distribution of the central compartment; Q, intercompartment clearance (L/h); V2, volume of distribution of the peripheral compartment; IIV, inter-individual variability; RSE, relative standard error; CI, confidence interval; R0, baseline response; K_out_, degradation rate constant; IC_50_, half-maximal inhibitory concentration; gamma, sigmoidicity of the drug effect.

**Table 4:** PBPK model predictions for time for which HOSU-53 trough concentrations in the liver remain above IC90 value from DHODH inhibition assay.

Dose (mg)	Average Time Above Target (h)	Median (h)	2.5th Percentile (h)	97.5th Percentile (h)
5	2.93	2.25	1	12.7
10	8.53	8	4	19.8
15	13.3	12.1	6	24
20	16.6	16.3	7.25	24
25	18.8	19.1	8.44	24

## Data Availability

The data presented in this study are available on request from the corresponding author. Restrictions may apply since this agent is entering clinical evaluation.

## Supplementary Materials

The following supporting information can be downloaded at: https://www.mdpi.com/article/10.3390/pharmaceutics17040412/s1, Figure S1: Goodness-of-fit plots of the final population PK mouse model. (a) Observed HOSU-53 concentration vs. population predicted and observed HOSU-53 concentration vs. individual predictions. (b) Individual-weighted residuals (IWRES) vs. time and individual predictions. (c) Prediction-corrected visual predictive check (pcVPC) for the final population PK model of mouse; Figure S2: Goodness-of-fit plots of the final population PD mouse models. (a) Observed DHO concentration vs. population predicted and observed DHO concentration vs. individual predictions. (b) IWRES vs. time and individual predictions. (c) PcVPC for the final population PD model of mouse; Figure S3: Normalized concentration-time profiles of different dose levels of HOSU-53 in rats (upper: normal scale, lower: log scale); Table S1: Overview of conducted PK/PD and toxicokinetic/toxicodynamic studies; Figure S4: Observed and PBPK model predicted plasma concentration of HOSU-53 in rat (a) after multiple doses of 3 mg/kg (sodium salt) PO administration and (b) after single dose of 1 mg/kg (lysine salt) oral administrations; Figure S5: Observed and PBPK model predicted plasma concentration of HOSU-53 in dog (a) after a single dose of 0.3 mg/kg (lysine salt) PO administration and (b) after multiple doses of 1 mg/kg (sodium salt) oral administrations; Figure S6: Predicted PK profiles of HOSU-53 in human using final PBPK rat model; Figure S7: Animal safety and efficacy correlate with DHO levels across multiple species; Table S2: Input parameters for the final rat PBPK model for HOSU-53; Table S3: PK parameter estimates of the final mouse PKPD model. For the determination of plasma HOSU-53/DHO concentrations, bioanalysis for all studies was conducted by Charles River Laboratories using ultra-high performance liquid chromatography with tandem mass spectrometry (UHPLC-MS/MS). Whole blood samples for HOSU-53/DHO were collected into tubes with K_2_EDTA. Tubes were stored on wet ice until processed to plasma by centrifugation (3500 rpm at 5 °C for 10 min) within 30 min of collection. Samples were then transferred into individual uniquely labeled matrix tubes and stored at nominal −70 ± 10 °C until analysis of HOSU-53 and DHO. Plasma concentrations of HOSU-53 and DHO were analyzed using 2 validated ultra-high performance liquid chromatography with tandem mass spectrometry (UHPLC-MS/MS) procedures in the electrospray ionization positive (ESI+) mode and electrospray ionization negative (ESI-) mode, respectively. The calibration curve concentration ranges were 10.0 to 5000 ng/mL for HOSU-53 and 10.0 to 30,000 ng/mL for DHO in mouse plasma. The calibration curve concentration ranges were 20.0 to 20,000 ng/mL for HOSU-53 and 50.0 to 5000 ng/mL for DHO in rat plasma. The calibration curve concentration ranges were 5.0 to 5000 ng/mL for HOSU-53 and 10.0 to 30,000 ng/mL for DHO in dog plasma. The calibration sample acceptance criteria were set to be within ± 15.0% (±20.0% at LLOQ). The incurred sample reanalysis was successfully conducted within the criteria, indicating that the bioanalytical assay used was reliable and reproducible.
