# Supplementary material for: Translational Pharmacokinetic-Pharmacodynamic Modeling of a Novel Oral Dihydroorotate Dehydrogenase (DHODH) Inhibitor, HOSU-53 (JBZ-001)
Source: Pharmaceutics. 2025 Mar 25;17(4):412. doi: 10.3390/pharmaceutics17040412 (PMC12030426; doi:10.3390/pharmaceutics17040412)

Supplementary Figure 1. Goodness-of-fit plots of the final population PK mouse model. (a) Observed HOSU-53 concentration vs. population predicted and observed HOSU-53 concentration vs. individual predictions. (b) Individual-weighted residuals (IWRES) vs. time and individual predictions. (c) Prediction-corrected visual predictive check (pcVPC) for the final population PK model of mouse.

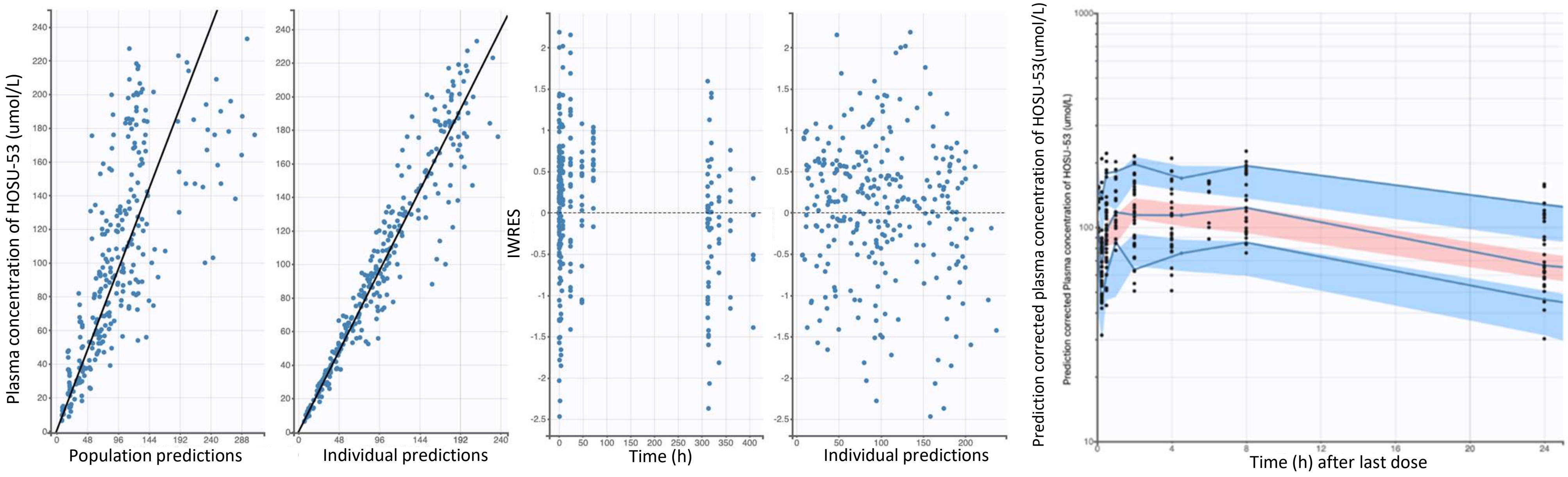

Supplementary Figure 2. Goodness-of-fit plots of the final population PD mouse models. (a) Observed DHO concentration vs. population predicted and observed DHO concentration vs. individual predictions. (b) IWRES vs. time and individual predictions. (c) PcVPC for the final population PD model of mouse.

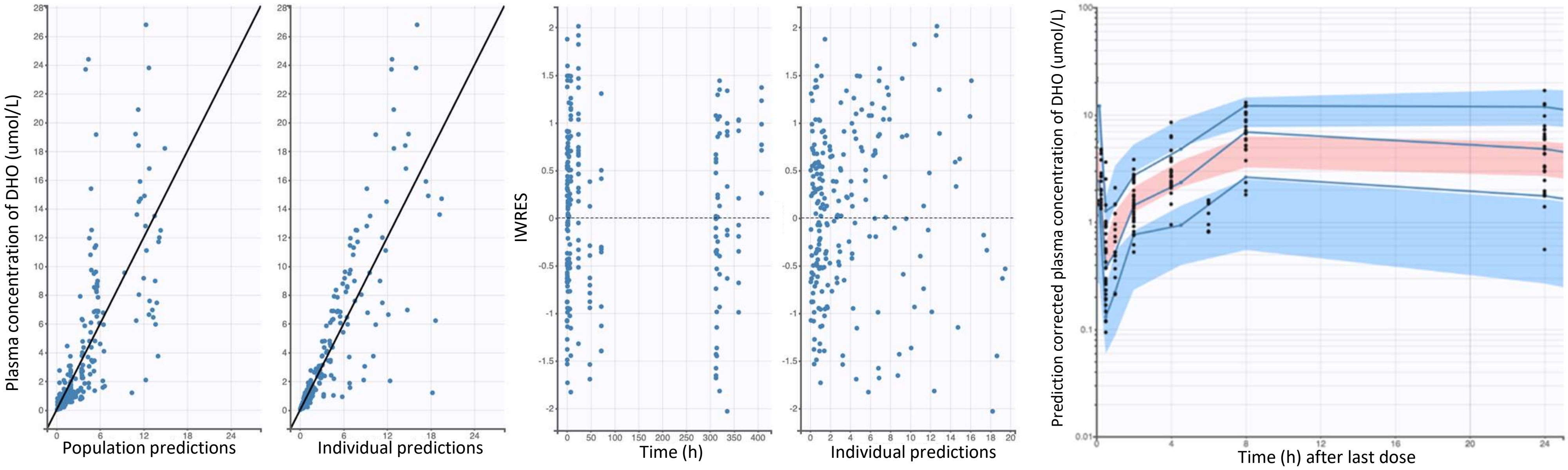

Supplementary Figure 3. Normalized concentration-time profiles of different dose levels of HOSU-53 in rats (left: normal scale, right: log scale)

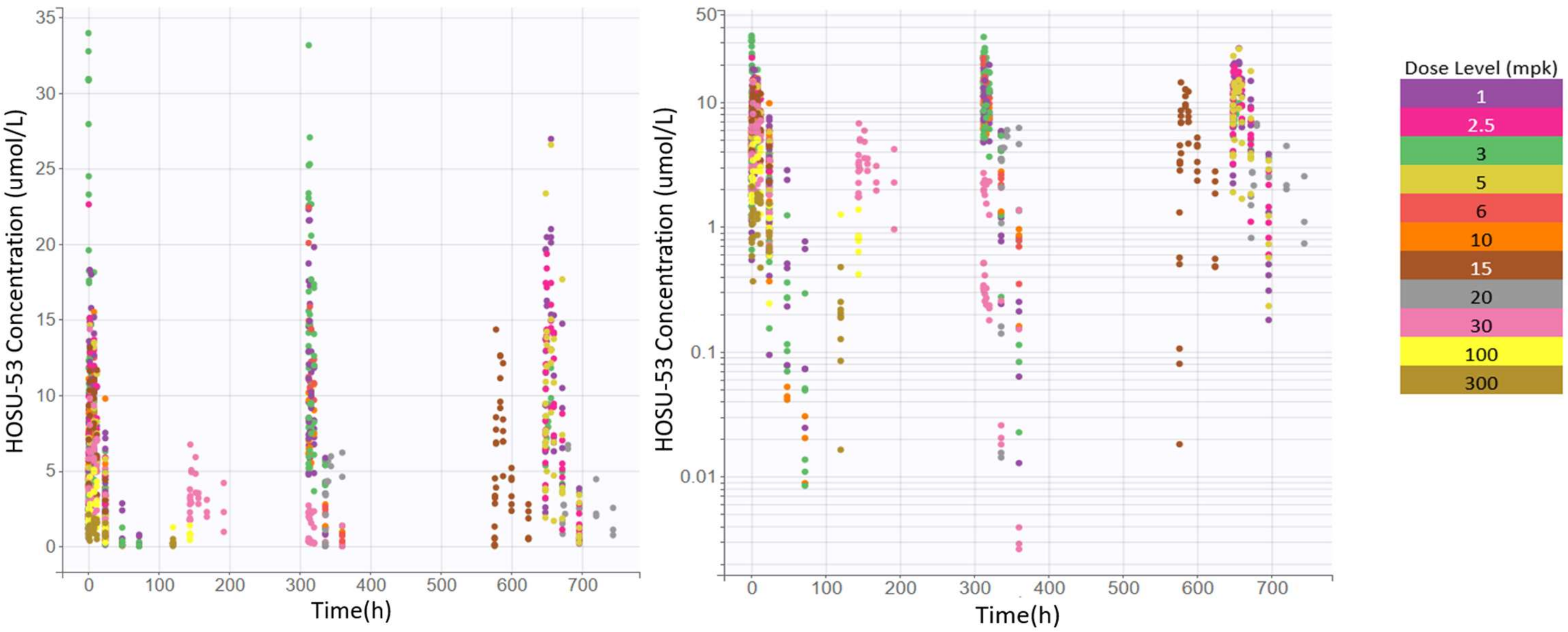

Supplementary Figure 4. Observed and PBPK model predicted plasma concentration of HOSU-53 in rat (a) after single dose of 1 mg/kg (lysine salt) oral administration and (b) after multiple doses of 3 mg/kg (sodium salt) oral administrations

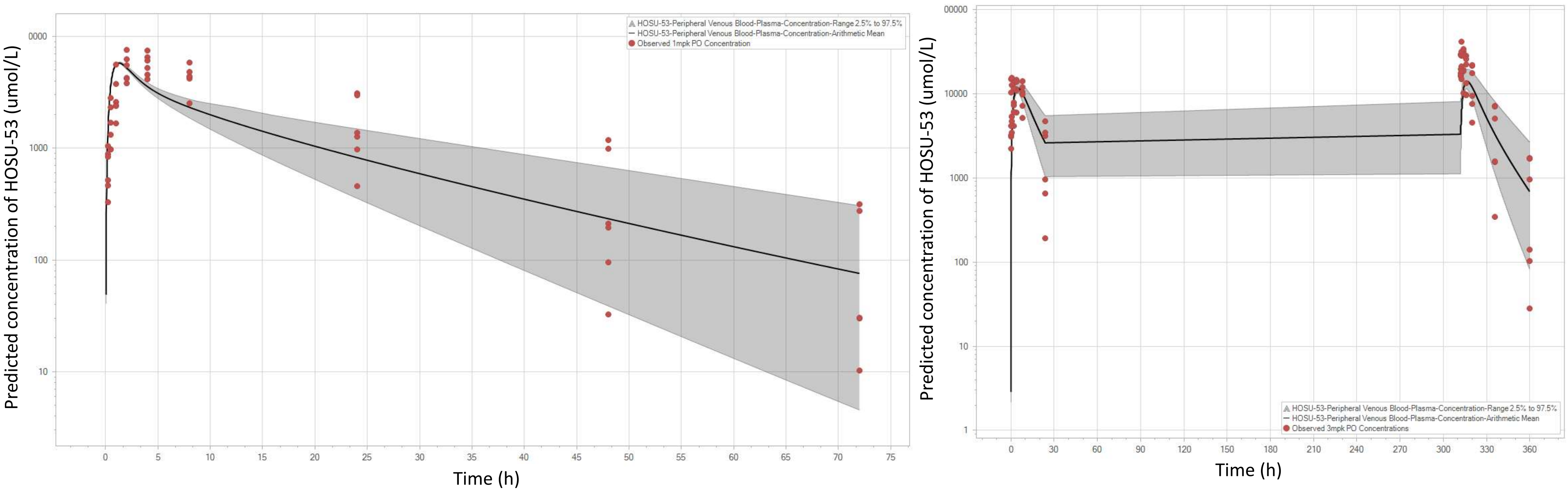

The red circles represent the measured plasma concentrations of HOSU-53. The solid line and grey-shaded area represent the model predicted mean concentration-time profiles and the 5th-95th percentile of the virtual rat population, respectively.

Supplementary Figure 5. Observed and PBPK model predicted plasma concentration of HOSU-53 in dog (a) after a single dose of 0.3 mg/kg (lysine salt) oral administration and (b) after multiple doses of 1 mg/kg (sodium salt) oral administrations.

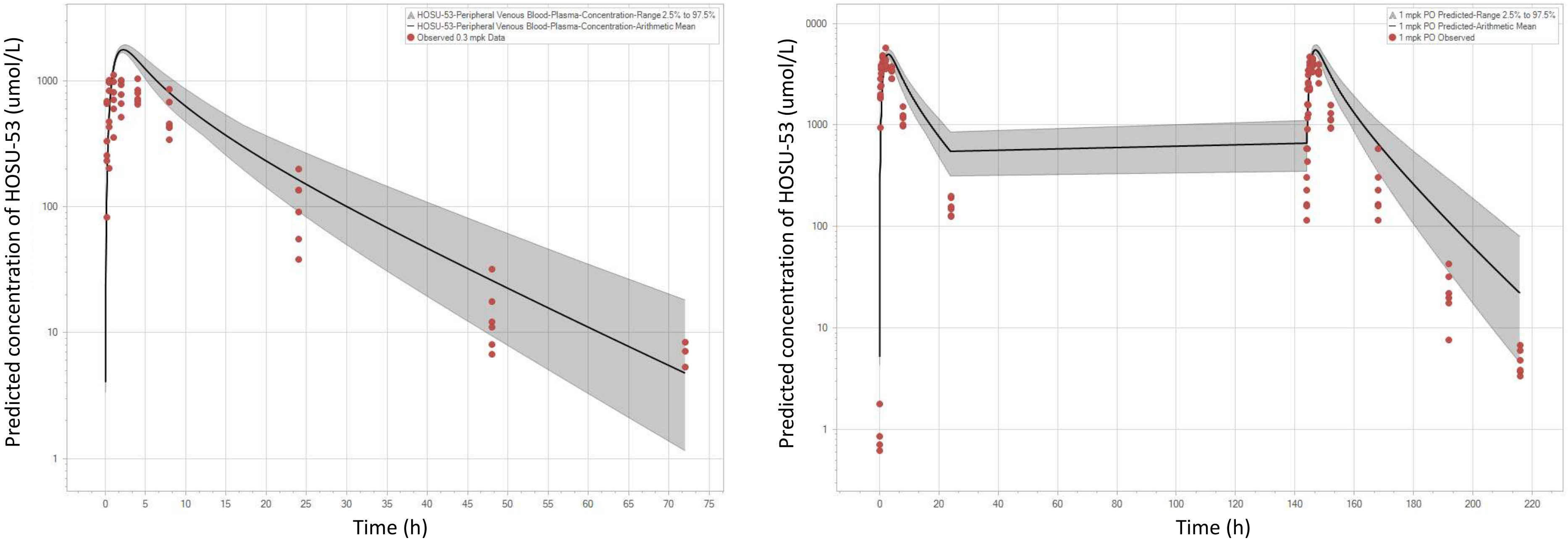

The red circles represent the measured plasma concentrations of HOSU-53. The solid line and grey-shaded area represent the model predicted mean concentration-time profiles and the 5th-95th percentile of the virtual dog population, respectively.

Supplementary Figure 6. Predicted PK profiles of HOSU-53 in human using final PBPK rat model

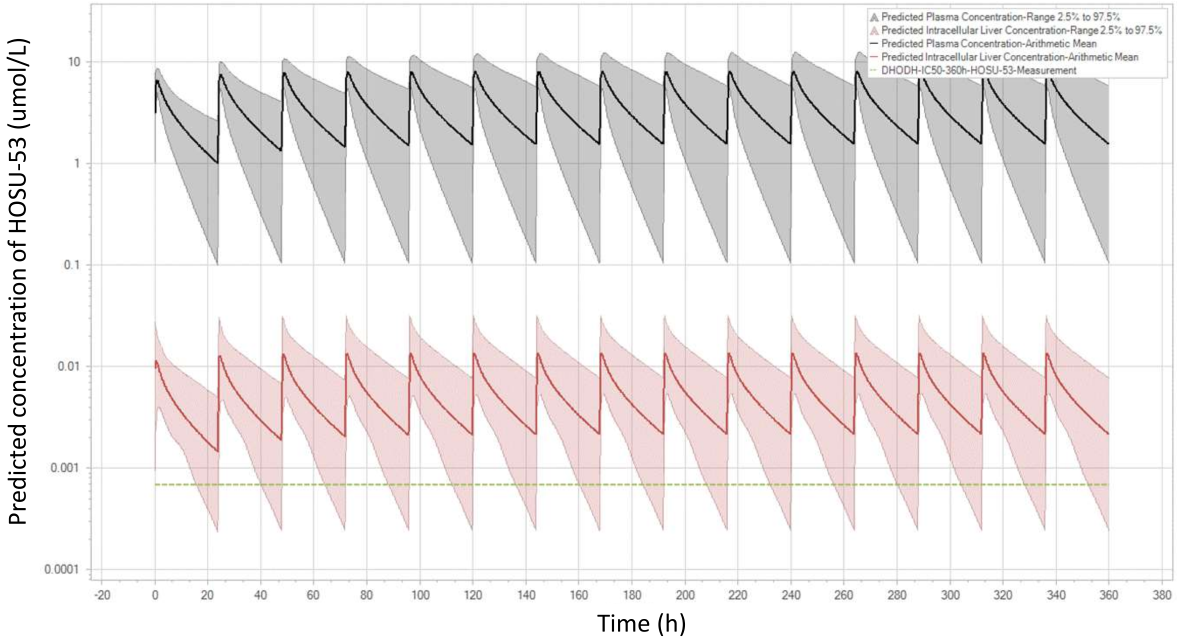

The black solid line and black shaded area represent mean predicted plasma HOSU-53 concentration-time profiles and 5th-95th percentile of the virtual human population. The red solid line and red shaded area represent mean predicted intracellular liver HOSU-53 concentration-time profiles and 5th-95th percentile of the virtual human population. The green dashed line represents measure IC_{50} value of HOSU-53 at 360 hours after dose administration.

Supplementary Figure 7. Animal safety and efficacy correlate with DHO levels across multiple species

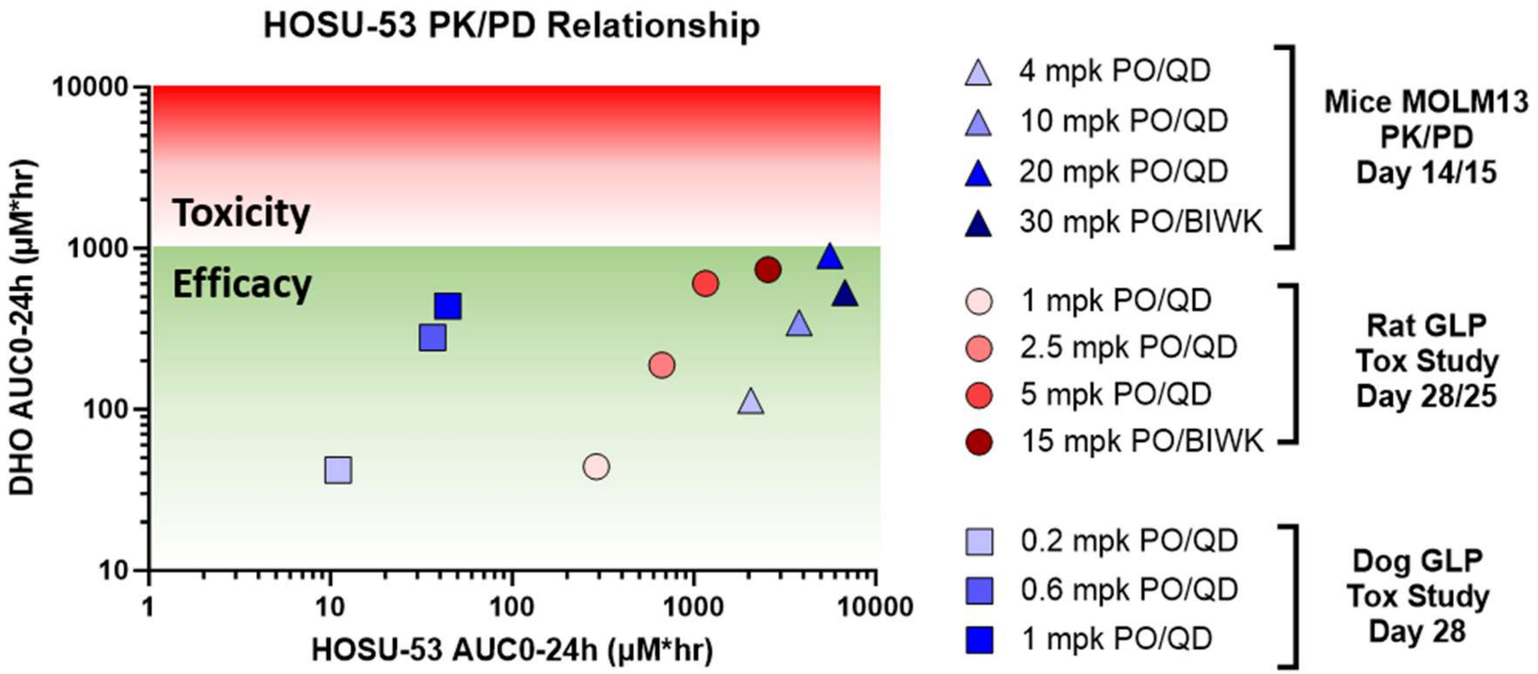

Supplement: Supplementary file 1 [file pharmaceutics-17-00412-s001.zip › SupplementaryFigures.pdf]
