# Supplementary material for: Translational Pharmacokinetic-Pharmacodynamic Modeling of a Novel Oral Dihydroorotate Dehydrogenase (DHODH) Inhibitor, HOSU-53 (JBZ-001)
Source: Pharmaceutics. 2025 Mar 25;17(4):412. doi: 10.3390/pharmaceutics17040412 (PMC12030426; doi:10.3390/pharmaceutics17040412)
Supplement: Supplementary file 1 [file pharmaceutics-17-00412-s001.zip › SupplementaryTable2.pdf]

Supplementary Table 2. Input parameters for the final rat PBPK model for HOSU-53

Parameter	Value (unit)	Source
Lipophilicity	4.35	Experimental data
Fupls	0.14%	Estimated in PKsim
pKa	1.19 (Base)	Experimental data
	3.05 (Acid)	Experimental data
Solubility	18.8 ug/ml (pH 6.5)	Experimental data
Intestinal Permeability (Sodium Salt – DI Water)	0.004 cm/min	Estimated in PKsim
Intestinal Permeability (Lysine Salt – DI Water)	0.07 cm/min	Estimated in PKsim
Specific Clearance	7.29 /min	Scaled from invitro hepatic stability assay
Blood:Plasma Concentration Ratio	0.59	Estimated in PKsim
