# Supplementary material for: Translational Pharmacokinetic-Pharmacodynamic Modeling of a Novel Oral Dihydroorotate Dehydrogenase (DHODH) Inhibitor, HOSU-53 (JBZ-001)
Source: Pharmaceutics. 2025 Mar 25;17(4):412. doi: 10.3390/pharmaceutics17040412 (PMC12030426; doi:10.3390/pharmaceutics17040412)
Supplement: Supplementary file 1 [file pharmaceutics-17-00412-s001.zip › SupplementaryTable3.pdf]

Supplementary Table 3. PK parameter estimates of the final mouse PKPD model

Parameters	Model estimates	Bootstrap result	
	Parameter estimates (RSE %)	Point estimate	95% CI
F (%)	0.53 (0.02)	0.54	0.44 – 0.61
Ka (/h)	1.0 (0.02)	1.2	0.72 – 1.2
CL/F (mL/h)	0.07 (5.0)	0.07	0.07 – 0.08
V1 (mL)	1.2 (8.7)	1.3	0.93 – 1.4
Q (mL/h)	1.6 (1.7)	1.6	1.2 – 2.2
V2 (mL)	1.2 (5.3)	1.1	1.0 – 1.4
IIV CL	0.32 (11.6)	0.32	0.24 – 0.35
IIV V1	0.53 (12.1)	0.49	0.40 – 0.66
IIV V2	0.16 (41.8)	0.28	0.07 – 0.31
Proportional residual error (PK)	0.18 (5.5)	0.18	0.14 – 0.21
R0 (umol/L)	0.01 (1.4)	0.01	0.004 - 0.02
Kout (/h)	155 (12.7)	127	55 - 260
IC50 (umol/L)	1.55 (8.9)	1.61	0.71 - 2.67
gamma	1.71 (0.1)	1.63	1.18 - 2.07
IIV Kout	0.62 (12.7)	0.64	0.41 - 0.89
IIV IC50	0.53 (8.9)	0.5	0.32 - 0.75
Proportional residual error (PD)	0.46 (6.3)	0.46	0.4 - 0.52

Abbreviations: F, bioavailability; Ka, first-order absorption rate constant; CL, clearance; V1, volume of distribution of the central compartment; Q, intercompartment clearance (L/h); V2, volume of distribution of the peripheral compartment; IIV, inter-individual variability; RSE, relative standard error; CI, confidence interval; R0, baseline response; Kout, degradation rate constant; IC50, half-maximal inhibitory concentration; gamma, sigmoidicity of the drug effect.
